# The effect of COVID-19 lockdown on admission rates in Maternity Hospital

**DOI:** 10.1515/med-2024-1062

**Published:** 2024-10-28

**Authors:** Jehad Abdullah Al-Harmi, Baydaa Alsannan, Fatemah Alhadhoud, Zahraa Akbar, Eman Alazmi, Khaled AlMuzayen, Eelaf Hussain, Mariam Aldarweesh, Basilio Pecorino, Antonio Simone Laganà, Antonio D’Amato, Vittorio Agrifoglio, Andrea Etrusco

**Affiliations:** Department of Obstetrics and Gynecology, College of Medicine, Kuwait University, Safat, 13110, Kuwait; Department of Obstetrics and Gynecology, Kuwait Ministry of Health, Maternity Hospital, Kuwait City, Kuwait; Postgraduate Training Program in Obstetrics and Gynecology, Kuwait Institute for Medical Specialization, Kuwait City, Kuwait; Obstetrics and Gynecology Division, Umberto I Hospital, Kore University of Enna, 94100, Enna, Italy; Unit of Obstetrics and Gynecology, “Paolo Giaccone” Hospital, Department of Health Promotion, Mother and Child Care, Internal Medicine and Medical Specialties (PROMISE), University of Palermo, 90127, Palermo, Italy; Department of Biomedical Sciences and Human Oncology, Obstetrics and Gynecology Unit, University of Bari “Aldo Moro”, 70124, Bari, Italy

**Keywords:** coronavirus, COVID-19, pandemic, early pregnancy complications, adverse health outcomes, obstetrics and gynecology emergencies, induction of labor

## Abstract

**Objectives:**

The COVID-19 pandemic had adverse health outcomes on individuals and communities. In this cross-sectional study we evaluated the admission rates in a tertiary-level hospital during the first wave of the pandemic (March 22, 2020 to August 31, 2020).

**Methods:**

We compared the indications for admission during the first wave of the pandemic to a control period prior to the lockdown (November 9, 2019 to March 22, 2020).

**Results:**

Most hospital admissions during the curfew period were obstetric emergencies (46.88%), which were significantly higher than the control group (38.19%) *p* ≤ 0.0001. Among the obstetric emergencies, cases in active labor (65.63%) were dominant. Significant rises in car deliveries (2.46%, *p* ≤ 0.0001) and admissions during the second stage of labor (6.43%, *p* ≤ 0.001) were noted. There was also an increased rate of admissions for early pregnancy complications, induction of labor, elective obstetric cases, and medical obstetric cases.

**Conclusions:**

This study demonstrates that lockdown precautions implemented had a significant impact on the rate of admissions to Maternity Hospital. The data obtained may be a used to aid in designing robust policies for future pandemics to avoid adverse health outcomes.

## 1 Introduction

The first reported cases of severe acute respiratory syndrome coronavirus 2 (SARS-CoV-2), the seventh human coronavirus (COVID-19), originated from Wuhan, China in January 2020 [1]. The WHO demonstrated that COVID-19 has a detrimental effect on social structure, economic advancement, and health services and systems. Numerous investigations revealed that the pandemic had a negative impact on maternity and newborn care services [2]. This is due to physiological changes in pregnancy that contribute to increased maternal vulnerability to maternal infections and mortality [3]. As a result, numerous countries attempted or initiated local and general lockdowns to limit the spread of the disease [4]. Lockdown protocols in public areas, workplaces, and healthcare facilities are strongly advised by the WHO [5]. In terms of healthcare services, some restricting measures were put in place to lower the quantity of in-person consultations. Two examples of telehealth were phone calls and virtual consultations [5,6]. Additionally, limiting the number of patients’ visitors and advising patients to visit the emergency room (ER) if necessary [6]. Routine antenatal care is a well-established priority; as a result instructions had been published by the health authorities not to disrupt the antenatal care during the pandemic [7]. However, the question remained whether the lockdown would negatively impact the quality of care because of reduced access to health services, including maternal and neonatal health services [7,8]. In addition, many pregnant women developed anxiety of acquiring the infection which prevented them further from visiting the hospital and seeking care [7].

Several studies have been conducted to estimate and assess the influence of the lockdown and hence reduced access to maternal health services on mortality rate [8]. The Guttmacher institute showed that as low as a 10% reduction in maternal and neonatal health services will possibly increase maternal and neonatal deaths globally up to 28,000 and 168,000 deaths, respectively [9]. Few studies illustrated an increase in the number of intrauterine fetal deaths (IUFD) and stillbirths during the pandemic, which could be due to reduced access to healthcare services [8]. Overall, the consequences of lockdown on maternal health have not been widely explored and further studies are required [9].

The Ministry of Health in Kuwait ordered lockdown, restricting travel, gatherings, and movement. Antenatal visits were limited in order to manage and stop the spread of COVID-19 and due to the shortage of medical staff who were self-isolated or quarantined.

In this study we aimed to assess and describe in detail the impact of COVID-19 lockdown on the rate of cases admitted to the Maternity Hospital and compare it with period before the lockdown. The major goals of this are to ascertain whether socioeconomic and demographic factors contribute to pandemics and to help formulate planning strategies for the future. In addition, exploring the modifications required to maternity health services in response to pandemics and other natural disasters in the future.

## 2 Methods

### 2.1 Study design

We conducted a cross-sectional analysis at a large tertiary centre. All women admitted during the lockdown period were included, and their admissions were compared with those prior the lockdown.

### 2.2 Study population

The two primary categories of women admitted to the Maternity Hospital for obstetrical or gynecological causes were included. All women admitted between November 9, 2019 and March 22, 2020 were involved in the control group; all women admitted between March 22 and August 31, 2020 were included in the lockdown group. The total curfew duration involved four main phases, which were determined by the Kuwaiti Cabinet. The strategy was to implement four phases: the first phase was a partial lockdown, which started from March 22, 2020 (5 pm to 4 am), the second phase was an extended partial curfew from April 24, 2020 (4 pm to 8 am), the third phase was a complete curfew from May 10, 2020, and the final phase was a partial curfew from May 31, 2020 (6 pm to 6 am).

**Figure 1:**
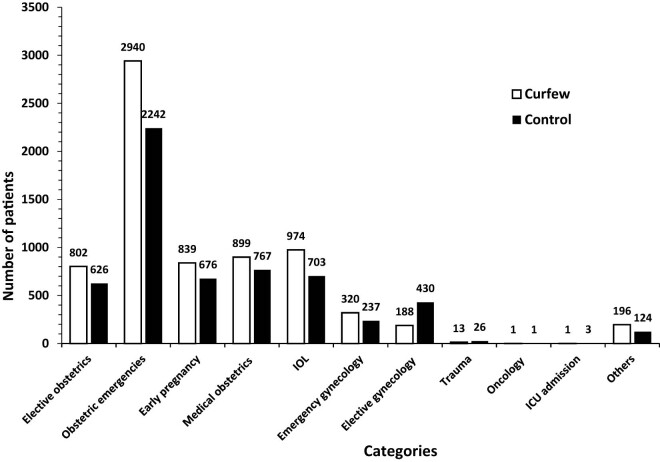
Overall admissions. Graph demonstrating overall admission rates during the COVID lockdown period and pre-COVID lockdown period. Admission categories included elective obstetrics, obstetrics emergencies, early pregnancy, medical obstetrics, (IOL), emergency gynecology, elective gynecology, trauma, oncology, (ICU) admission and others.

**Figure 2:**
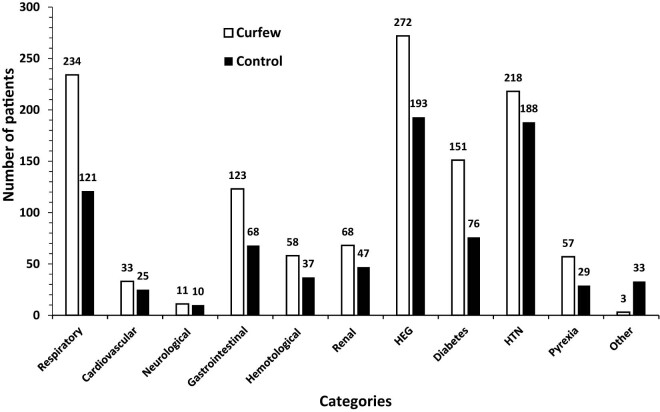
Medical obstetrical admissions. Medical obstetric admissions subdivided into the following categories; respiratory, cardiovascular, neurological, gastrointestinal, hematological, renal, hyperemesis gravidarum (HEG), diabetes in pregnancy, hypertensive (HTN) disorders of pregnancy, pyrexia and others.

**Figure 3:**
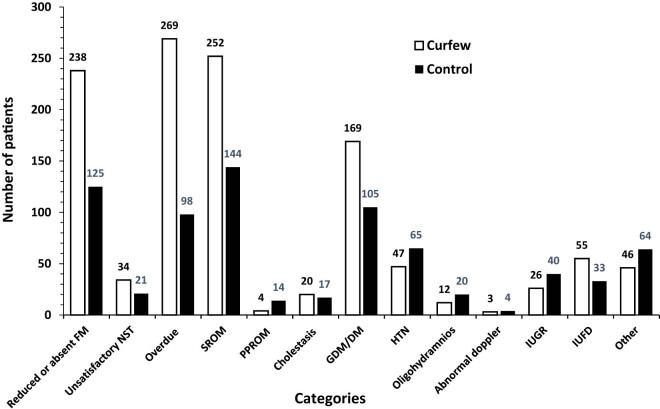
Admissions for induction of labor. IOL admissions were divided into the following reasons; reduced or absent fetal movement (FM), unsatisfactory (NST), overdue, spontaneous rupture of the membrane (SRM), (PPROM), intrahepatic cholestasis of pregnancy, gestational diabetes (GDM) or pre-existing diabetes, oligohydramnios, abnormal fetal Doppler studies, (IUGR), (IUFD), and others.

**Figure 4:**
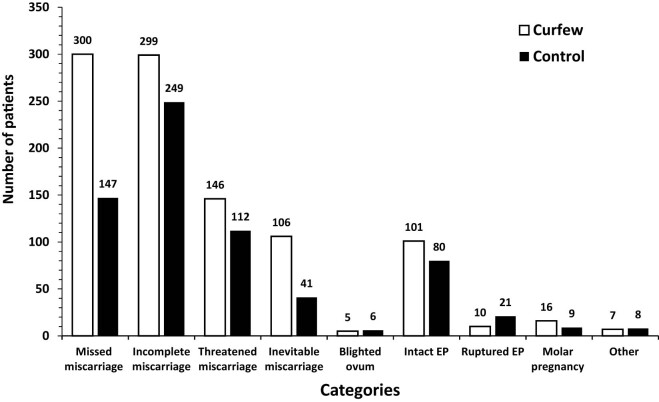
Admissions for early pregnancy complications. Early pregnancy complications admissions divided into missed, incomplete, threatened and inevitable miscarriage, blighted ovum, intact or ruptured ectopic pregnancy (EP), molar pregnancy and others.

**Figure 5:**
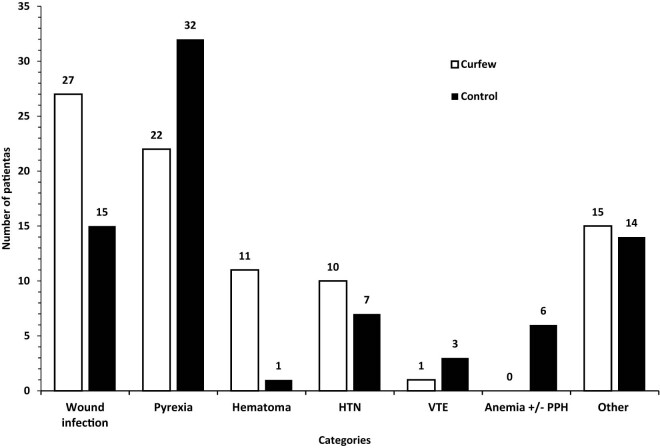
Postpartum admissions. Postpartum admissions were divided into wound infection, pyrexia, hematoma, hypertension (HTN), venous thromboembolism (VTE), anemia with or without postpartum hemorrhage (PPH), and others.

A systematic, structured questionnaire completed by designated authors for data collection was used to obtain data from hospital records. Eleven main categories were identified on the admission indicator (Figure 1). Cervical cerclage and elective cesarean section fell under the heading of elective obstetric procedures. A number of conditions were included in the emergency obstetrical category: active labor, preterm labor (PTL), rupture of the membranes (ROM), intrauterine growth restriction (IUGR), abnormal fetal Doppler studies, oligohydramnios, polyhydramnios, unsatisfactory non-stress test, diminished fetal movements, and antepartum hemorrhage. The subcategory of “active labor” was further divided based on the stage of labor (first or second stage) and the place of delivery (home, automobile, or ER). The medical obstetric cases were sub-categorized according to the main complaint (Figure 2). The induction of labour (IOL) category was divided according to the indication (Figure 3). Three subcategories were identified for the early pregnancy cases: molar pregnancy, ectopic pregnancy, and miscarriage (Figure 4). The following categories applied to emergency gynecology cases: heavy menstrual bleeding (HMB), adnexal torsion, tubo-ovarian abscess, genital abscess, pelvic inflammatory disease, and ovarian hyperstimulation syndrome. Elective gynecology cases were divided into ovarian cyst with or without cystectomy, fibroid surgery, polypectomy, total abdominal hysterectomy with or without bilateral salpingo-oophorectomy, diagnostic dilatation and curettage, hysteroscopy, bilateral tubal ligation, urogynecology surgery, endometriosis, and adnexal mass. The postpartum cases were divided into wound infection, pyrexia, hematoma, postpartum hypertension, venous thromboembolism, and anemia (Figure 5). The categories of trauma cases included assault, falls from heights, and traffic accidents.

The primary goal was to determine the Maternity Hospital’s admissions rate during the pandemic’s initial wave and compare it with the admissions rate before the outbreak.

### 2.3 Statistical analysis

Data entry was carried out using Excel Software and analyzed using analysis of variance NOVA). The descriptive statistics were reported as mean ± standard deviation. The categorical variables were reported as numbers and percentages. Chi-square test and Fisher Exact test were used. *p-*Value ≤ 0.05 was considered significant.


**Ethical approval:** The study was approved by institutional Ethics Committee of the Ministry of Health (2020/1494).

## 3 Results

We evaluated 6,272 ER admissions throughout the partial and total curfew study period and compared the indications of admission to the 5,870 admissions during the lockdown-free control period.

The majority of admissions fall within the category of obstetric emergencies in both periods (46.88% in curfew and 38.19% in control, OR = 1.43; 95% CI: 1.33–1.4; *p* ≤ 0.0001). On the other hand, the oncology category had the least admissions with a total of one admission for both periods.

Additional admission indicators including, IOL, medical obstetrics, early pregnancy complication, and elective obstetrics were 15.53, 14.33, 13.38, and 12.79% in curfew versus 11.98, 13.07, 11.52, and 10.66% in control group (OR, 95% CI and *p*-values of 1.35, 1.22–1.50, ≤0.0001; 1.11, 1.00–1.24, 0.05; 1.19, 1.07–1.32, 0.002; 1.23, 1.10–1.37, 0.001) (Figure 1).

Out of the total number of admissions, during both periods, majority of patients hospitalized were in labor (65.63% in curfew and 61.75% in control). SRM was the second highest indication in both periods, 17.31% during curfew and 13.85% during control period. PTL accounted for 11.94 and 11.97% during the curfew and control periods, respectively.

In “active labor” emergency obstetric cases, most patients presented in the first stage of labor (93.99% curfew vs 95.46% control). The remaining patients, 6.43% in curfew and 3.76% in control group, were admitted in the second stage of labor. Car deliveries went up to 2.46% during the curfew period as opposed to 0.23% during the control period. Furthermore, there was a rise in home births during the curfew period (0.58 vs 0.31% during the control period). Both periods’ ER deliveries were comparable.

A higher number of patients were admitted for medical obstetric indications during the curfew, 899 compared to 767 patients, *p* ≤ 0.05. Most of which were admitted for vomiting or hyperemesis gravidarum, during both the curfew (29.66%) and control periods (24.49%). Respiratory symptoms were another admission indicator that significantly increased throughout the curfew period, rising to 25.52% during the curfew compared to 15.36% in the control group. During the curfew period, 6.22% of patients were admitted with pyrexia, compared to 3.68% during the control period (Figure 2).

During the curfew, 58.31% of patients were admitted from the ER for IOL, compared to 41.68% in the control group. The strongest indication for IOL was overdue which accounted 27.39% in curfew group and 13.96% in control period. Another common indication was reduced fetal movement; 24.24% compared to 17.81% in curfew and control periods, respectively. There were a higher number of cases admitted for IOL due to ROM during the curfew (25.66%) compared to the control period (20.51%). IOL for IUFD was also increased during the curfew (*n* = 55, 5.6%) compared to the control period (*n* = 33, 4.7%) (Figure 3).

There were fewer trauma cases hospitalized during the curfew (12 vs 26 cases) in comparison to the control period. The most common cause of injury was falls from heights, with six cases during the curfew and fourteen during the control period. Throughout the curfew period, there was a decrease in the number of admissions resulting from traffic accidents: 8.33% compared to 26.92% in the control group. Admissions due to physical assaults were also lower in the curfew group as opposed to the control group (2 vs 4 cases, respectively) (Figure 1).

The vast majority of obstetrical elective admissions were elective CS. 89.5% of curfew participants had elective CS, compared to 94.3% in the control period (*p* = 0.01). Cervical cerclage was the second most frequent reason for an elective obstetrical admission (9.8% in curfew vs 3.7% in control group; *p* = 0.69).

Admissions for early pregnancy complications were dominated by cases of miscarriage. Miscarriage (including blighted ovum, inevitable, missed, and incomplete miscarriage) accounted for 71.7% of the cases in curfew group and 65.8% of control group; *p* = 0.04. Cases of ectopic pregnancy represented 11.2% in curfew period versus 15% in control group, with *p* = 0.05 (Figure 4).

In terms of gynecological elective admissions, there were more cases prior to the curfew (194 vs 495). Ovarian cyst complications were most frequently reported during the curfew, accounting for 29.9% of cases, compared to 17.2% prior to it (*p* = 0.15). Remarkably, during the curfew period, admissions for D&C reached 25.3% as elective gynecological admissions, compared to 3.4% during the control period (*p* = 0.16). In both groups, HMB was the most common emergency gynecological admission.

## 4 Discussion

The present study highlights the impact of lockdown during COVID-19 pandemic on the rate of hospital admissions in a tertiary care center, Maternity Hospital, in comparison with the pre-pandemic period. Overall, the admission rates were higher during the lockdown period as compared to the control period, 51.65 and 48.34%, respectively. Obstetric emergency accounted for 46.88% of admissions throughout the curfew period. The increase could be explained by the fact that most patients had difficulty getting outpatient healthcare services during the pandemic and that close patient monitoring is required, which is impractical in an ER. In a multicenter study conducted in Italy the admission rates were reduced by greater than 50% during the COVID-19 lockdown period [10]. It has been proposed that this reduction was not dependent on COVID-19 incidence, but rather on the lockdown itself [10]. Moreover, a 42% reduction in admission rates during the COVID-19 lockdown period was noted in a general hospital in USA and this could be explained by patients avoiding hospital visits to reduce their risk of acquiring COVID-19 [11]. Another retrospective study conducted in Italy demonstrated 35.4% decrease in ER admissions [12]. Interestingly, an increase in obstetric conditions hospitalization of 28% was noticed during the lockdown period and it has been proposed that this could be due to the lack of availability of contraceptive measures and the absence of proper transportation, which resulted in delayed obstetrician’s visits [13]. Kilfoyle et al. showed that 35.6% of pregnant women utilized the ER for non-emergent indications [14]. One study proposed several factors that could explain this finding such as difficulties in getting an outpatient appointment [15]. Use of the ER for non-indicated conditions can lead to unnecessary testing and treatment [16]. This also may explain the reason behind an increase in rate of hospital admissions in this study. Two policies will be discussed in this study. They are believed to have been crucial in the observed results (Table 1). A significant rise in car deliveries (2.46%, *p* ≤ 0.0001) and admission in the second stage of labor (6.43%, *p* ≤ 0.001) were noted in the curfew period. This could be explained by the lockdown restriction rules. A retrospective cohort study assessed how the pandemic affected the rates of admission to the delivery room, in comparison to the control period, there was a marked rise in urgent obstetrical conditions to the ER during the pandemic, with a higher percentage of pregnant women presenting in active labor (9.3%) and premature rupture of membrane (PROM) (20.6%) [7]. It has been explained by the way pregnant women’s altered behavior during the pandemic caused them to arrive late to the ER than expected. Also, pregnant women with confirmed COVID-19 infection had a higher risk of miscarriage, pre-eclampsia, PTL, and cesarean delivery [17,18]. This might contribute to the overall increase in urgent obstetrical conditions presenting to the ER during the pandemic when compared to the control period. That said, pregnant women also avoid presenting to the obstetrical ER as much as possible. Another study compared the number of pregnant women presented to the obstetric ER during the pandemic to their controls; the number of pregnant women presented to the obstetric ER in the matched period 1 year before the pandemic, and it has been noticed that the overall proportion of pregnant women presented and admitted during the pandemic were less compared to their control, and the most common reason for admission during the pandemic was active labor and PROM [7]. The present study showed a significant rise in home childbirth during the lockdown period. This has not yet justified and could be explained as a result of travel restrictions during lockdown. A delay in the management of high-risk pregnancies may have contributed to the increased incidence of pregnancy’s complications [18–20]. Hence more cases of IUFD, IUGR, PPROM, oligohydramnios, PTL, and reduced fetal movements have been reported as short-term consequences [21]. Moreover, this policy contributed to a higher admission rate in the IOL category, mainly due to overdue indication. One study observed an increase in the rate of IUFD which is consistent with the present study [12]. This could be due to the lack of outpatient follow up, which resulted in poor patient education and underestimation of the important warning signs of IUFD such as reduced fetal movements. We compared our findings to those of a systematic review and meta-analysis conducted in 2021. Similar to their results, we found an increased rate of IOL for IUFD which indicates an increase in stillbirths during the curfew period. Furthermore, in accordance with Ministry of Health standards at the time, we did not admit any COVID-19 positive patients, so there were no cases of maternal death in our hospital. The rate of preterm delivery was unchanged as reported in that review [22]. Although there is no current evidence that pregnant women are more susceptible to COVID-19 infection, nor that pregnant women infected with COVID-19 are at higher risk for severe pneumonia [23], it has been reported that viral pneumonia in pregnant women was associated with higher rates of PTL, IUGR, and perinatal mortality [23]. Our study showed that the admission rates due to medical complications in pregnancy, early pregnancy complications mainly missed abortion, and gynecological emergency conditions including HMB with or without blood transfusion, were increased during the curfew period, although the results were not statistically significant. This could be also explained by the first policy. Interestingly, elective obstetrical admissions were found to be higher during the lockdown period and the reason behind this is believed to be due to the second policy. Also, elective D&C for missed miscarriage were found to be higher during the lockdown period. A retrospective study conducted in Italy illustrated similar findings with an increase in the admission rate of elective cesarean section and IOL conditions from 47.5% in 2019 to 53.6% in 2020 [12]. Regarding trauma cases, it is observed that the rate of trauma admissions reduced during the lockdown period. During the curfew, there is a decrease in the number of admissions for physical assaults and traffic accidents. It is stated that the significant reduction in worldwide trauma cases was a direct effect of lockdown and reduced vehicular movement due to lockdown enforced by most countries [15]. With regards to gynecologic cases in the current study, a statistically significance rise of emergency cases (5.1%, *p* ≤ 0.006), and drop of elective cases (3%, *p* ≤ 0.0001), during the curfew period were noticed. Grandi et al. found a similar result stating that COVID-19 lockdown reduced the rate of admission for gynecological indications [10]. Moreover, another retrospective study conducted in Italy demonstrated that the admissions to the ER were reduced by 35.4% during the lockdown period, including a significant reduction for gynecological conditions such as genital infections [12]. These findings were similar to this current study for pelvic inflammatory disease and genital abscess conditions, but not for tubo-ovarian abscess cases. Most of the recorded accesses were referable to HMB due to uterine pathology [24–26]. Interestingly, however, training programs [27] and new technologies [28,29] aimed at investigating and treating the causes of HMB have not stopped, but indeed have progressed to establish themselves stronger than ever and expanding its limits to boundaries once unthinkable [30–32] at the end of the health emergency. As for data on reproductive medicine these are more difficult to come by; however, data on the consumption of drugs and supplements aimed at improving infertility would seem to show that this branch of the discipline is only partially affected [33].

**Table 1:** Ministry of Health policies during COVID-19 pandemic lockdown

Policy	
Policy I	Reduce the capacity and number of patients per clinic with lowering the time taken with each patient and establishing virtual OPD for remote follow-up
Policy II	Only cesarean section and dilatation and curettage conditions were allowed to be conducted in the elective surgical list

Comparable to prior studies, this research has a number of limitations involving human errors. A good example for this is a misunderstanding and misinterpretation of the data collected from handwritten admission logs. Moreover, data were entered using Survey Monkey questionnaire by different data collectors and as a result inter-observer errors may have occurred. The other limitation in our study is the categorization of the admission indications that was developed by the data collectors, which may have led to missing or redundant data. Additionally, the results may be inapplicable to the general population as this is a single-center, cross-sectional study.

This study also has multiple strengths including a large sample size for both COVID-19 lockdown and control periods. Furthermore, the findings of the study may aid in preparing the hospitals and government in facing of similar situations in the future.

In summary, this study was conducted with the assumption that the lockdown will be an obstacle faced by patients and doctors. From the patient’s perspective, the study proposed that patients might experience difficulty accessing healthcare facilities earlier and thus the rate of complications and emergency cases would increase during the lockdown period. However, from the physician’s side, concerns of discharging moderate risk patients from the ER during the lockdown period might have inflated the rate of hospital admissions. Finally, the increase in ER visits during a pandemic requires good structural preparation with the implementation of protocols and well-trained personnel [34].
